# Essential cell-intrinsic requirement for GMDS in T cell development

**DOI:** 10.3389/fimmu.2025.1598923

**Published:** 2025-06-26

**Authors:** Mehmet Yabas, Carla M. Roots, T. Daniel Andrews, Matt A. Field, Christopher C. Goodnow, Anselm Enders

**Affiliations:** ^1^ Division of Immunology and Infectious Disease, The John Curtin School of Medical Research, The Australian National University, Canberra, ACT, Australia; ^2^ Department of Immunology, Faculty of Medicine, Malatya Turgut Ozal University, Malatya, Türkiye; ^3^ Centre for Tropical Bioinformatics and Molecular Biology, College of Science and Engineering, James Cook University, Cairns, QLD, Australia; ^4^ The Garvan Institute of Medical Research, Sydney, NSW, Australia; ^5^ Cellular Genomics Futures Institute, School of Biomedical Sciences, University of New South Wales, Sydney, NSW, Australia

**Keywords:** fucosylation, glycosylation, GMDS, immune system, T cells, T cell development

## Abstract

Fucosylation, a type of glycosylation, is the attachment of a fucose to *N*-glycans, *O*-glycans and glycolipids, and is critical for the post-translational regulation of many essential pathways. Here we describe a mouse strain with an *N*-ethyl-*N*-nitrosourea-induced point mutation in the gene encoding guanosine diphosphate (GDP)-mannose 4,6-dehydratase (GMDS), an enzyme involved in the generation of GDP-fucose, a substrate for fucosylation. Gmds^Y187*/Y187*^ mice displayed growth retardation and increased postnatal mortality. Immunophenotyping of Gmds^Y187*/Y187*^ mice revealed reduced numbers of double positive (DP), CD4 single positive (SP) and CD8SP T cells, despite normal numbers of double negative (DN) cells in the thymus of mutant animals. Similarly, analysis of the thymus in *Rag1^-/-^* mice reconstituted with Gmds^Y187*/Y187*^ bone marrow cells revealed a partial arrest at the DN stage of T cell development compared to animals transplanted with *Gmds^+/+^* bone marrow cells. Furthermore, mixed chimeras showed that Gmds^Y187*/Y187*^ T cells were unable to compete with *Gmds^+/+^* cells from the DP stage of T cell development in the thymus. This inability to compete resulted in the near absence of Gmds^Y187*/Y187*^-derived peripheral T cells in recipient mice, while B cell subsets were present at broadly normal frequencies. These findings provide the first evidence of an essential cell-intrinsic requirement for GMDS in early T cell development in mice.

## 1 Introduction

Glycosylation is the attachment of sugars to proteins and is an important post-translational modification mechanism that affects and regulates the structure and biological function of the target proteins (1). It is estimated that more than half of all proteins undergo glycosylation (2). Fucosylation is a type of protein glycosylation in which a fucose is attached to the amino group of an asparagine in the endoplasmic reticulum (ER) (*N*-fucosylation) or to the hydroxyl group of a serine or threonine in the ER, Golgi, cytosol and nucleus (*O*-fucosylation) (3, 4). A nucleotide-charged form of fucose, known as guanosine diphosphate (GDP)-fucose, is used as the substrate to modify the target proteins in mammals (3, 4). GDP-fucose is generated in the cytoplasm by two pathways, the *de novo* synthesis pathway and the salvage pathway (3, 4). In the first step of the *de novo* pathway, GDP-4-keto-6-deoxy-mannose is synthesized from GDP-mannose and this process is catalyzed by an enzyme called GDP-mannose 4,6-dehydratase (GMDS) (5, 6). In the second step, the conversion of GDP-4-keto-6-deoxy-mannose to a GDP-fucose is catalyzed by the GDP-4-keto-6-deoxymannose-3,5-epimerase-4-reductase (FX) protein (7). The *de novo* pathway is thought to account for 90% of GDP-fucose synthesis (8, 9). In contrast, in the salvage pathway, GDP-fucose is generated by a GDP-mannose-independent pathway from free fucose available from dietary sources or lysosomal degradation of fucosylated proteins (3). GDP-fucose generated by either the *de novo* or the salvage pathway is then transported to the ER and Golgi where it is used by fucosyltransferases (FUTs) in the formation of fucosylated glycans (10).

Fucosylation of proteins is important in a variety of biological processes, including host-microbe interaction, selectin-dependent leukocyte adhesion, the immune system and cancer, largely through genetic ablation experiments, and dysregulation has been shown in several pathological conditions (4, 10, 11). For example, loss of the FX protein, which catalyzes the final step of GDP-fucose synthesis, resulted in a complete loss of cellular fucosylation, which was associated with embryonic lethality, reduced weight gain and postnatal survival, and increased neutrophil numbers (12). The latter phenotype in FX^-/-^ mice was shown to be regulated by Notch-dependent signaling (13). In addition, animals deficient in some FUTs (FUT8, FUT12/POFUT1 and FUT13/POFUT2) have been reported to exhibit developmental abnormalities (14–16). In the immune system, fucosylation is critical for selectin-mediated leukocyte extravasation and lymphocyte homing. In humans, a deficiency of the *SLC35A* gene, which encodes the enzyme responsible for the transfer of GDP-fucose to the Golgi, results in a defect in leukocyte rolling (17–20). Antibody-mediated cellular cytotoxicity has been shown to be increased by 50-100-fold in the absence of core fucosylation of IgG1 (21, 22). Moreover, *O*-fucosylation of Notch family receptors is essential for activation of the Notch signaling pathway, which plays a critical role in the immune system (14, 23). Using FUT8-deficient mice, core fucosylation has been shown to be important for T cell development and activation (24–26), and to enhance anti-tumor immune responses of T cells by reducing PD-1 expression (27, 28). In this study, we report the immunological effects of an *N*-ethyl-*N*-nitrosourea-induced mutation in *Gmds*, which encodes an enzyme used in the first step of the *de novo* GDP-fucose synthesis pathway, and demonstrate that GMDS is critical for normal T cell development in mice.

## 2 Materials and methods

### 2.1 Mice

Gmds^Y187*/Y187*^ (*Gmds^m1Anu/m1Anu^*) mice were generated by *N*-ethyl-*N*-nitrosourea mutagenesis, and the causal mutation was mapped using a previously published strategy (29). For the mapping process, an unaffected carrier was bred with a CBA mouse, and the resulting offspring were randomly intercrossed. The F2 mice were phenotyped, and all the mice from breeder pairs that produced affected offspring were genotyped for a panel of markers evenly distributed across all chromosomes. This defined an area on Chromosome 13, between rs13481734 and rs3701164, that was associated with the phenotype.

In parallel, we performed exome sequencing on an affected mouse to identify homozygous variants, as previously described (30). The only protein changing variant identified in the interval was the GMDS^Y187*^ mutation. To eliminate any unrelated mutations, the line was maintained for more than ten generations by crossing a Gmds^Y187*/+^ carrier with a wild-type C57BL/6J mouse. Experimental mice were generated by crossing heterozygous Gmds^Y187*/+^ mice from the backcross. PCR amplification was performed from DNA isolated from mouse-ear punches for genotyping. All experimental mice were housed under specific pathogen-free conditions at the Australian National University, and all animal procedures were approved by the Australian National University Animal Ethics and Experimentation Committee under protocols A2014/62 and A2017/54.

### 2.2 Flow cytometry

Flow cytometric analysis of cell suspensions from bone marrow, thymus and spleen was performed as previously described (30, 31). Briefly, single cell suspensions were prepared and red blood cells were lysed for spleen samples. For surface staining, cells were counted using the ViCELL cell counter (Beckham Coulter, Brea, CA, USA) and equal numbers of cells were first treated with purified rat anti-mouse CD16/CD32 (BD, San Jose, CA, USA; clone 2.4G2) and anti-biotin antibodies in ice-cold staining buffer (phosphate-buffered saline containing 2% bovine serum and 0.1% NaN_3_) and incubated for 30 min at 4°C in the dark. Cells were then washed and stained with a primary antibody cocktail containing an appropriate combination of the antibodies and streptavidin in ice-cold staining buffer and incubated for 30 min at 4°C in the dark. Samples were washed with staining buffer and resuspended in the same buffer, followed by analysis on an LSR II (BD, San Jose, CA, USA) or LSR Fortessa flow cytometer (BD, San Jose, CA, USA) and FlowJo 887 software (FlowJo, LLC, Ashland, OR, USA) was used for data analysis.

The following antibodies were used for flow cytometry analysis: anti-CD3 Alexa Fluor 700 (eBioscience, San Diego, CA, USA; clone 145-2C11), anti-CD4 APC (BD, San Jose, CA, USA; clone RM4-5), anti-CD4 APC Cy7 (BioLegend, San Diego, CA, USA; clone GK1.5), anti-CD8a PE (BD; clone 53-6.7), anti-CD8a PE Cy7 (Biolegend; clone 53-6.7), anti-CD11b APC Alexa Fluor 750 (eBioscience; clone M1/70), anti-CD19 Brilliant Violet 510 (Biolegend; clone 6D5), anti-CD19 APC Vio770 (Miltenyi Biotec, Bergisch Gladbach, Germany; clone 6D5), anti-CD21/35 FITC (BD; clone 7G6), anti-CD23 Pacific Blue (Biolegend; clone B3B4), anti-CD23 PE (BD; clone B3B4), anti-CD25 Biotin (BD; clone 7D4), anti-CD25 FITC (BD; clone 7D4), anti-CD44 Pacific Blue (Biolegend; clone IM7), anti-CD45R/B220 APC (BD; clone RA3-6B2), anti-CD45R/B220 AF700 (BD; clone RA3-6B2), anti-CD45R/B220 Brilliant Violet 605 (Biolegend; clone RA3-6B2), anti-CD45.1 Alexa Fluor 700 (Biolegend; clone A20), anti-CD45.1 FITC (BD; clone A20), anti-CD45.2 APC (eBioscience; clone B104), anti-CD45.2 Pacific Blue (Biolegend; clone B104), CD62L Brilliant Violet 605 (Biolegend; clone MEL-14), anti-CD93 Biotin (eBioscience; AA4.1), anti-Gr-1 Biotin (BD; clone RB6-8C5), anti-IgD FITC (eBioscience; clone 11-26c), anti-IgM PE Cy7 (BioLegend; clone 53-6.7), anti-NK1.1 PE (BD; clone PK136), Streptavidin Brilliant Violet 605 (Biolegend), Streptavidin PE Cy7 (eBioscience), anti-TCRβ APC Cy7 (Biolegend; clone H57-597), anti-TCRγδ FITC (BD; clone GL3) and 7-aminoactinomycin D (Thermo Fisher Scientific, Waltham, MA, USA).

### 2.3 Bone marrow chimera

100% chimeras were generated using bone marrow cells extracted and counted from *Gmds^+/+^* and Gmds^Y187*/Y187*^ donor mice and injected intravenously into *Rag1* ^-/-^ mice (32) that were irradiated with a single dose of 500 rads. Mixed chimeras were generated using bone marrow cells extracted from either *Gmds^+/+^* CD45.2 or Gmds^Y187*/Y187*^ CD45.2, counted and mixed 1:1 with *Gmds^+/+^* CD45.1/2 animals. *Gmds^+/+^* CD45.1 recipient mice were irradiated with two doses of 450 rads prior to intravenous injection of bone marrow donor cells for each recipient mouse. The second cohort of mixed chimeras was generated by extracting, counting, and mixing 1:1 bone marrow cells from either *Gmds^+/+^* CD45.2 or Gmds^Y187*/Y187*^ CD45.2 with *Gmds^+/+^* CD45.1 animals. *Rag1* ^-/-^ recipient mice were irradiated with a single dose of 500 rads prior to intravenous injection of with bone marrow donor cells for each recipient mouse.

### 2.4 Statistical analysis

Data were statistically analyzed using GraphPad Prism 9 for Mac OS X (GraphPad Software, San Diego, CA, USA). Student’s *t*-test was used to compare only two groups. When multiple experimental groups were compared, one-way ANOVA with Tukey’s multiple comparison test was used. For all statistical analysis, differences were considered to be significant when *p* < 0.05.

## 3 Results

### 3.1 Discovery of an *N*-ethyl-*N*-nitrosourea-induced Gmds^Y187*/Y187*^ mouse strain with growth retardation and poor survival

**Figure 1:**
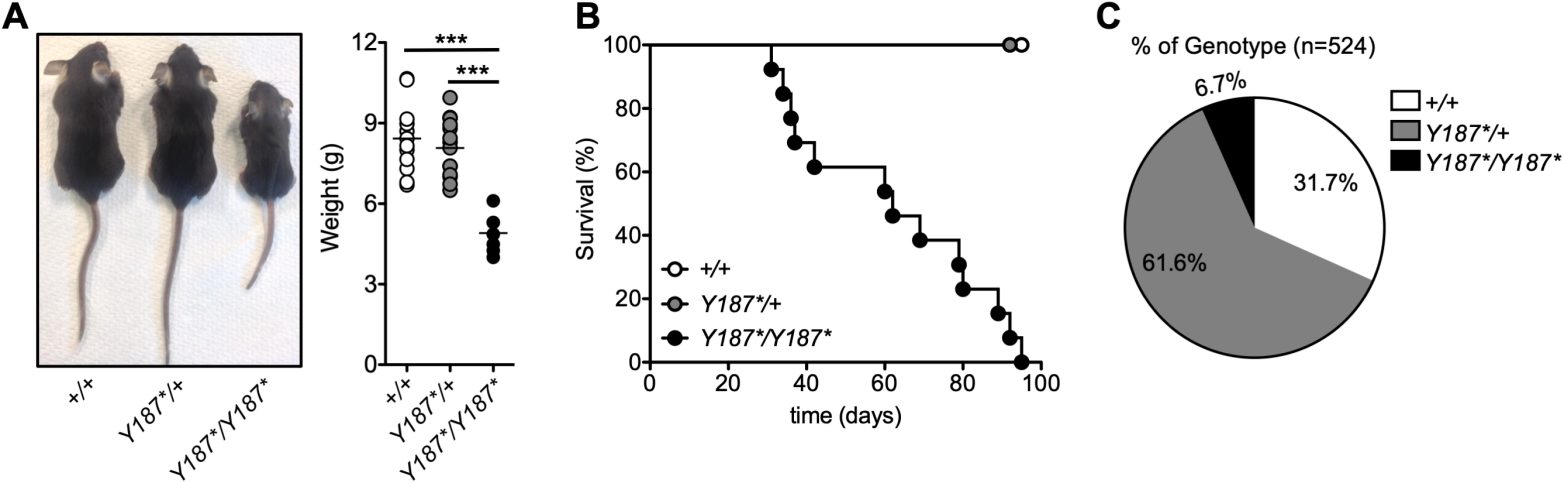
Discovery of an *N*-ethyl-*N*-nitrosourea-induced Gmds^Y187*/Y187*^ mouse strain with growth retardation and poor survival. **(A)** Gross appearance of *Gmds^+/+^*, Gmds^Y187*/+^ and Gmds^Y187*/Y187*^ mice at 3 weeks of age. The graph shows the body weight of *Gmds^+/+^*, Gmds^Y187*/+^ and Gmds^Y187*/Y187*^ mice at 3 weeks of age. Each symbol represents an individual mouse and n=12 for *Gmds^+/+^*, n=14 for Gmds^Y187*/+^ and n=7 for Gmds^Y187*/Y187*^ pooled from five independent experiments. **(B)** Kaplan–Meier survival plot shows postnatal survival of *Gmds^+/+^* (n=20), Gmds^Y187*/+^ (n = 20) and Gmds^Y187*/Y187*^ (n=18) littermates. **(C)** The pie chart shows the percentage of live genotypes born from the Gmds^Y187*/+^ x Gmds^Y187*/+^ cross (n=524). Statistical analysis in **(A)** was performed using one-way ANOVA followed by Tukey’s post-test, with *p* values comparing each pair of experimental groups shown in the graph. ***p < 0.001.

To identify novel genes with critical roles in the development and/or function of immune cells (29), we screened mice carrying *N*-ethyl-*N*-nitrosourea-induced point mutations. Among the offspring, several mice exhibited severe growth retardation after weaning and a reduction in body weight of approximately 42% (**Figure 1A**). The causative mutation was mapped to the gene encoding GMDS, which catalyzes the conversion of GDP-mannose to GDP-4-keto-6-deoxymannose, the first step in the synthesis of GDP-fucose from GDP-mannose, which can be used as a substrate for fucosylation (5). The mutation is a T-to-A substitution at position 818 on cDNA that results in the conversion of tyrosine to a STOP codon at amino acid 187 (p.Y187*). The mutation is predicted to remove the last 185 amino acids of GMDS, potentially leading to loss of cellular fucosylation. The median survival of mice with the growth defect was 5 weeks and all Gmds^Y187*/Y187*^ mice died by 95 days after birth (**Figure 1B**). Genotyping of offspring from intercrosses of Gmds^Y187*/+^ mice showed that *Gmds^+/+^* and Gmds^Y187*/+^ mice were born at the expected 1:2 ratio but less than 7% of all weaned mice were homozygous (**Figure 1C**), suggesting the requirement for proper fucosylation for embryonic development or survival in the early postnatal phase before weaning (14–16). However, Gmds^Y187*/+^ mice were virtually indistinguishable from their *Gmds^+/+^* littermates in terms of size and survival (**Figures 1A, B**).

### 3.2 A partial developmental arrest at the double negative stage of T cell development in the thymus of Gmds^Y187*/Y187*^ mice

**Figure 2:**
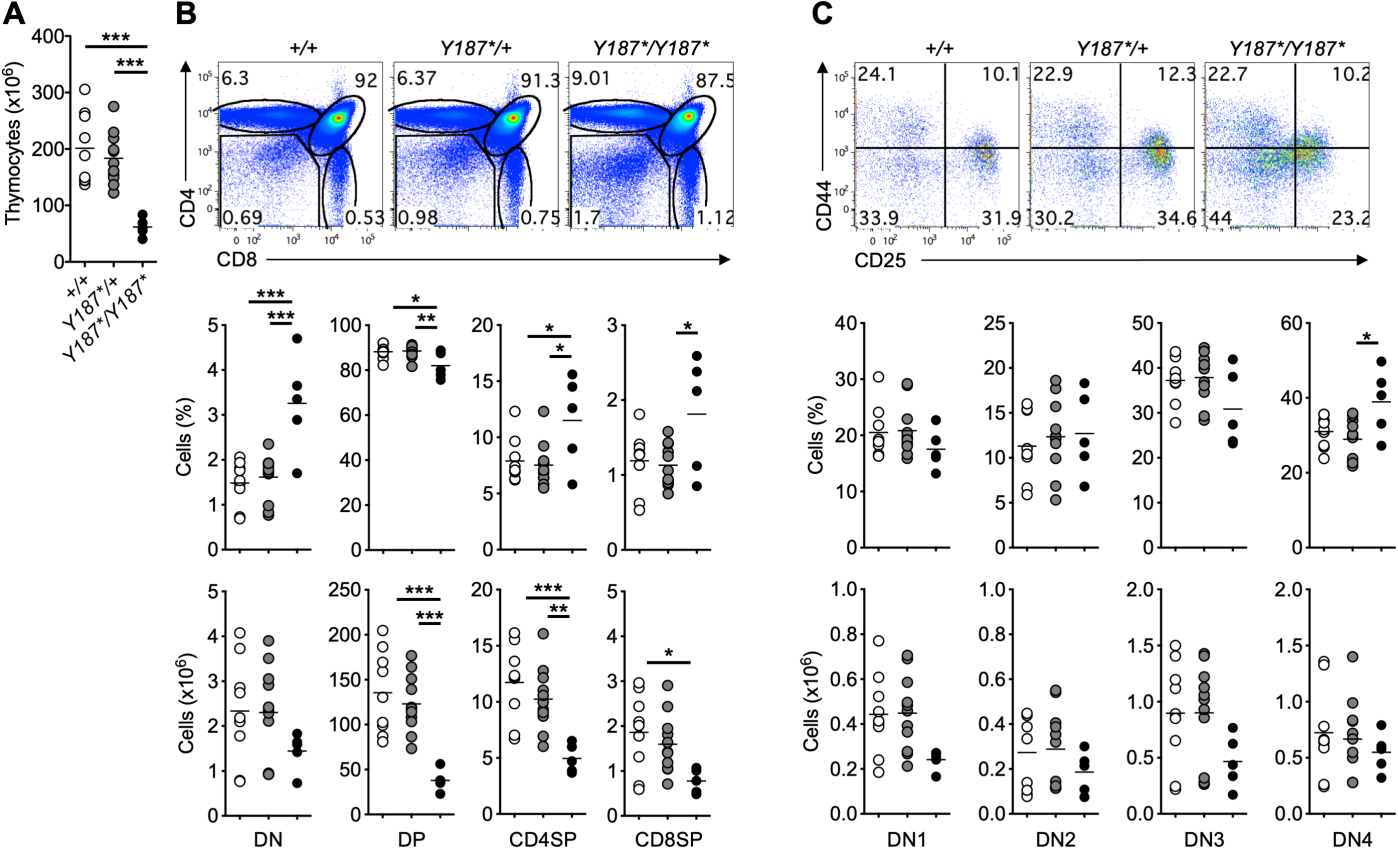
Partial developmental arrest at the DN stage of T cell development in the thymus of Gmds^Y187*/Y187*^ mice. **(A)** The graph shows the absolute number of total thymocytes in *Gmds^+/+^*, Gmds^Y187*/+^ and Gmds^Y187*/Y187*^ mice at 3 weeks of age. **(B)** Representative flow cytometric analysis of T cell development in the thymus of *Gmds^+/+^*, Gmds^Y187*/+^ and Gmds^Y187*/Y187*^ mice. The graphs show percentage and absolute number of different T cell subsets in *Gmds^+/+^*, Gmds^Y187*/+^ and Gmds^Y187*/Y187*^ mice at 3 weeks of age. **(C)** Representative flow cytometric analysis of DN T cell subsets in the thymus of *Gmds^+/+^*, Gmds^Y187*/+^ and Gmds^Y187*/Y187*^ mice based on their CD44 and CD25 expression. The graphs show the percentage and absolute number of different DN T cell subsets in *Gmds^+/+^*, Gmds^Y187*/+^ and Gmds^Y187*/Y187*^ mice at 3 weeks of age. Each symbol in **(A–C)** represents an individual mouse, and n=9 for *Gmds^+/+^*, n=11 for Gmds^Y187*/+^ and n=5 for Gmds^Y187*/Y187*^ pooled from four independent experiments. Statistical analysis was performed using one-way ANOVA, followed by Tukey’s post-test, with *p* values comparing each pair of experimental groups shown in the graphs. ***p < 0.001; **p < 0.01; *p < 0.05.

While the reduced survival could be attributed to the requirement for fucosylation during embryonic and/or postnatal development (14–16), we focused our investigation on the role of GMDS in lymphocyte development. As we observed the death of Gmds^Y187*/Y187*^ mice at approximately 5 weeks of age, we immunophenotyped the mice at 3 weeks of age by analyzing the thymus and spleen. Gmds^Y187*/Y187*^ mice had lower thymic cellularity compared to their Gmds^Y187*/+^ and *Gmds^+/+^* littermates (**Figure 2A**). Analysis of different T cell subsets in the thymus of Gmds^Y187*/Y187*^ mice at 3 weeks of age revealed a small but statistically significant decrease in the frequency of double positive (DP) cells with a concomitant increase in the percentage of DN cells, CD4 single positive (SP) and CD8SP cells (**Figure 2B**). In absolute terms, this phenotype resulted in normal numbers of DN thymocytes, but significantly reduced numbers of DP, CD4SP and CD8SP cells (**Figure 2B**). This observation is consistent with the up-regulation of core fucosylation at the transition from DN to DP cells in the thymus (26).

Depending on the expression of the adhesion molecule CD44 and the interleukin (IL)-2 receptor α chain CD25, DN cells can be divided into four subpopulations, namely CD44^+^CD25^–^ DN1 cells, CD44^+^CD25^+^ DN2 cells, CD44^–^CD25^+^ DN3 cells, and CD44^–^CD25^–^ DN4 cells (33). Detailed analysis of the DN subsets based on their CD44 and CD25 expression revealed a lower CD25 expression on Gmds^Y187*/Y187*^ thymocytes (**Figure 2C**). This resulted in a trend towards a reduced percentage of DN3 cells and increased DN4 cells in the thymus of Gmds^Y187*/Y187*^ mice (**Figure 2C**). However, the absolute numbers of different DN subsets in the thymus of Gmds^Y187*/Y187*^ mice appeared normal compared to their Gmds^Y187*/+^ and *Gmds^+/+^* littermates (**Figure 2C**). Interestingly, reduced expression of CD25 was specific to DN cells, with CD4^+^FoxP3^+^ cells in the thymus and spleen of Gmds^Y187*/Y187*^ mice exhibiting comparable levels of CD25 expression to those of Gmds^Y187*/+^ and *Gmds^+/+^* (**Supplementary Figure 1**). These data suggest that the mutation in the gene encoding GMDS affects the development of T cells in the thymus.

### 3.3 Normal T cell numbers in the spleen of Gmds^Y187*/Y187*^ mice

**Figure 3:**
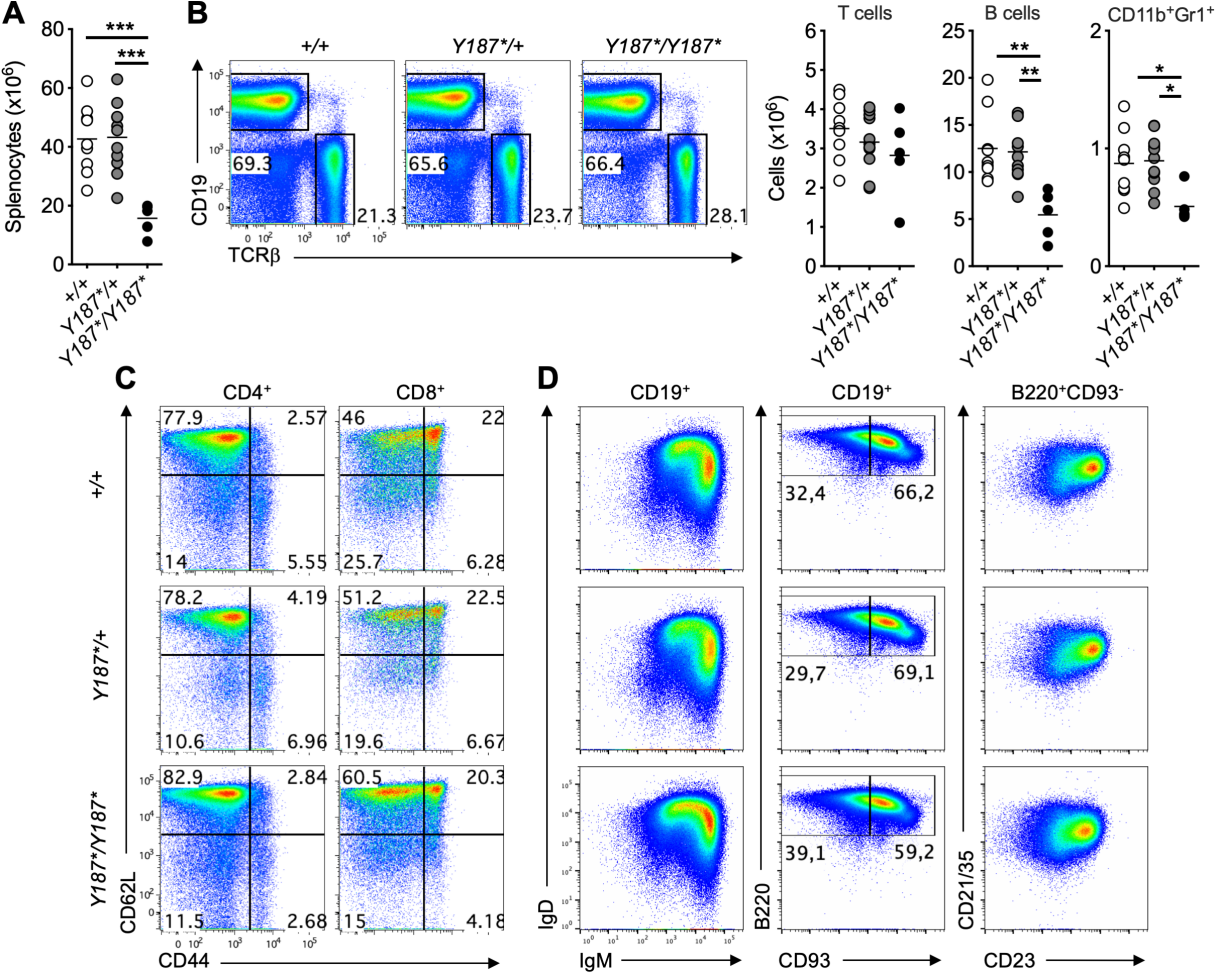
Normal presence of T cells in the spleen of Gmds^Y187*/Y187*^ mice at 3 weeks of age. **(A)** The graph shows the absolute number of total splenocytes in *Gmds^+/+^*, Gmds^Y187*/+^ and Gmds^Y187*/Y187*^ mice at 3 weeks of age. **(B)** Representative flow cytometric analysis of CD19 and TCRβ in the spleen of *Gmds^+/+^*, Gmds^Y187*/+^ and Gmds^Y187*/Y187*^ mice. The graphs show the absolute number of T cells, B cells and CD11b^+^Gr1^+^ myeloid cells in the spleen of *Gmds^+/+^*, Gmds^Y187*/+^ and Gmds^Y187*/Y187*^ mice at 3 weeks of age. **(C)** Representative flow cytometric analysis of CD62L vs CD44 expression on CD4^+^ (left) and CD8^+^ (right) T cells in the spleen of *Gmds^+/+^*, Gmds^Y187*/+^ and Gmds^Y187*/Y187*^ mice. **(D)** Representative flow cytometric analysis of IgD vs IgM expression (left) or B220 vs CD93 expression (middle) on CD19^+^ B cells, and CD21/35 vs CD23 expression (right) on B220^+^CD93^-^ mature B cells in the spleen of *Gmds^+/+^*, Gmds^Y187*/+^ and Gmds^Y187*/Y187*^ mice. Gates were set based on an adult *Gmds^+/+^* mouse analyzed at the same time. Each symbol in **(A, B)** represents an individual mouse, and n=9 for *Gmds^+/+^*, n=11 for Gmds^Y187*/+^ and n=5 for Gmds^Y187*/Y187*^ pooled from four independent experiments. Statistical analysis was performed using one-way ANOVA, followed by Tukey’s post-test, with *p* values comparing each pair of experimental groups shown in the graphs. ***p < 0.001; **p < 0.01; *p < 0.05.

We then examined the different immune cell subsets in the spleen of mice. At 3 weeks of age, the absolute number of total splenocytes in Gmds^Y187*/Y187*^ mice was less than 50% of their Gmds^Y187*/+^ and *Gmds^+/+^* littermates (**Figure 3A**). Surprisingly, despite the fact that Gmds^Y187*/Y187*^ mice had a partial developmental block at the DN stage of T cell development in the thymus (**Figure 2**) and that the total number of splenocytes was significantly reduced, Gmds^Y187*/Y187*^ mice had comparable number of T cells in the spleen (**Figure 3B**). Analysis of CD62L and CD44 expression on CD4^+^ and CD8^+^ T cells also revealed a largely normal profile in the spleen of Gmds^Y187*/Y187*^ mice compared to their Gmds^Y187*/+^ and *Gmds^+/+^* littermates (**Figure 3C**; **Supplementary Figure 2**).

The total numbers of B cells and CD11b^+^Gr1^+^ myeloid cells were significantly reduced in the spleen of Gmds^Y187*/Y187*^ mice (**Figure 3B**). Detailed analysis revealed the normal presence of B cell subsets in the spleen of Gmds^Y187*/Y187*^ mice (**Figure 3D**; **Supplementary Figure 3**). As Gmds^Y187*/Y187*^ mice had essentially normal splenic B cell subsets, the reduction in total B cells and CD11b^+^Gr1^+^ numbers could simply be attributed to either the lower total number of splenocytes in Gmds^Y187*/Y187*^ mice (**Figure 3A**) or an unknown effect of the GMDS^Y187*^ mutation on the development and/or survival of other immune cell subsets, including innate lymphoid cells (ILCs).

### 3.4 GMDS acts cell-intrinsically to regulate T cell development

**Figure 4:**
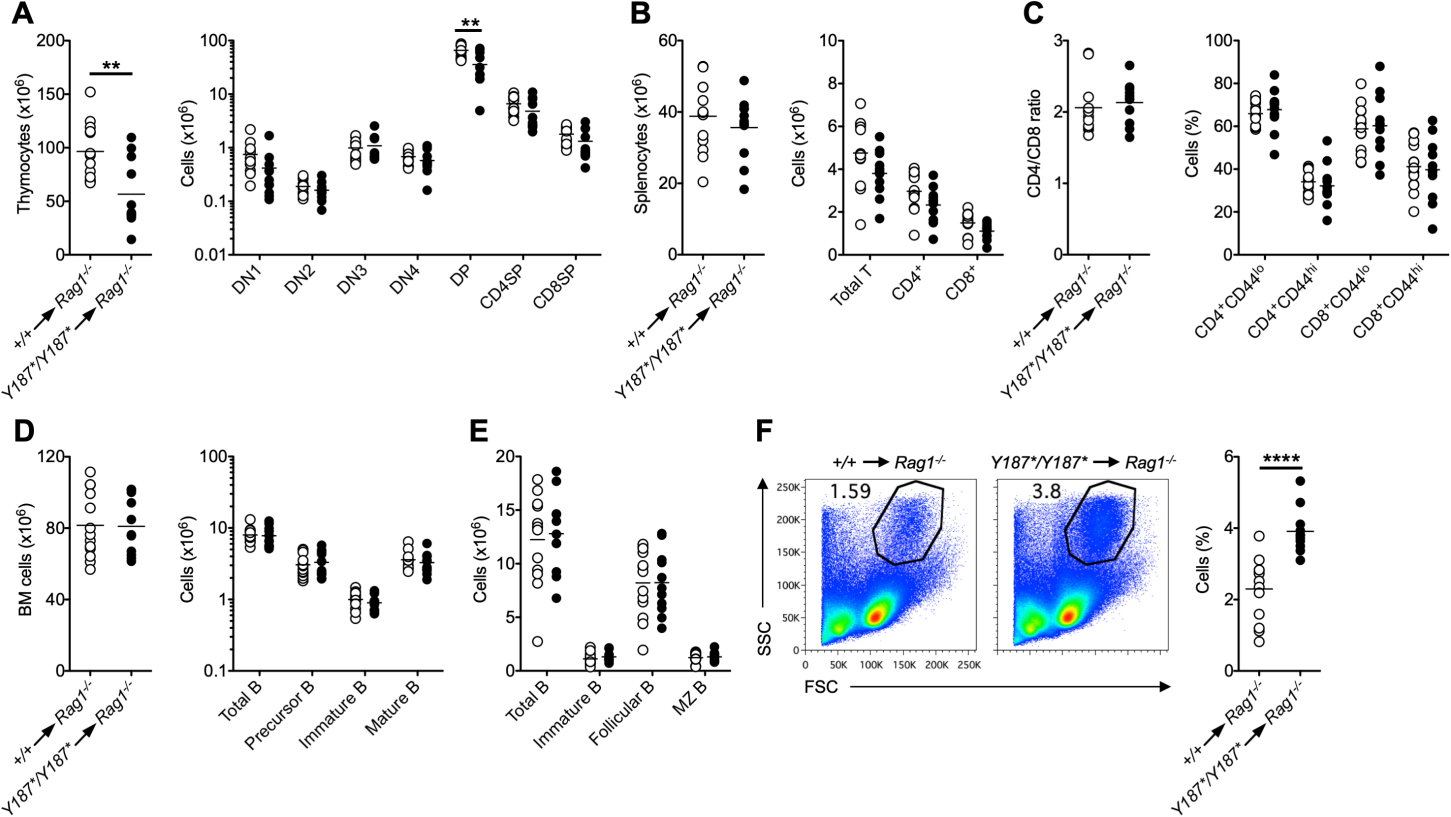
GMDS acts cell-intrinsically to regulate T cell development. Bone marrow cells from either *Gmds^+/+^* or Gmds^Y187*/Y187*^ mice were transferred into irradiated *Rag1^-/-^* animals followed by flow cytometric analysis after reconstitution. **(A)** The graphs show the absolute numbers of total thymocytes (left) and different T cell subsets (right) in the thymus of recipient mice. **(B)** The graphs show the absolute numbers of total splenocytes (left) and total T, CD4^+^ T and CD8^+^ T cells (right) in the spleen of recipient mice. **(C)** The graphs show the ratio between CD4^+^ T and CD8^+^ T cells (left) and the percentage of CD44^hi^ and CD44^lo^ cells within CD4^+^ and CD8^+^ T cells (right) in the spleen of recipient mice. **(D)** The graphs show the absolute numbers of total bone marrow cells (left) and total B, IgM^-^IgD^-^ precursor B, IgM^+^IgD^-^ immature B and IgM^+^IgD^+^ mature B cells within the B220^+^ gate (right) in the bone marrow of recipient mice. **(E)** The graph shows the absolute numbers of total B, immature B, follicular B cells and marginal zone (MZ) B cells in the spleen of recipient mice. **(F)** Representative flow cytometric analysis of the SSC vs FSC profile in the spleen of recipient mice. The graph shows the percentage of cells. Each symbol represents an individual recipient mouse, and n=13 for *Gmds^+/+^* and n=11 for Gmds^Y187*/Y187*^ pooled from two independent experiments analyzed 65 days and 160 days post-transplantation. Statistical analysis was performed using Student’s *t*-test for each cell population shown in the graphs. **p < 0.01; ****p < 0.0001.

Since Gmds^Y187*/Y187*^ mice showed poor postnatal survival and a partial block in T cell development (**Figures 1**, **2**), we next wanted to determine whether the T cell phenotype observed in intact mice resulted from lack of GMDS-mediated fucosylation in hematopoietic cells. To this end, we performed bone marrow transplantation, where *Gmds^+/+^* or Gmds^Y187*/Y187*^ bone marrow cells were transferred into irradiated lymphoid-deficient recipients (*Rag1^-/-^*) followed by analysis after reconstitution. Consistent with the analysis of intact Gmds^Y187*/Y187*^ mice, these experiments showed that the total number of thymocytes was significantly reduced in *Rag1^-/-^* mice transplanted with Gmds^Y187*/Y187*^ bone marrow cells compared to *Rag1^-/-^* mice transplanted with *Gmds^+/+^* bone marrow cells (**Figure 4A**). While the number of DN cell subsets in mice transplanted with Gmds^Y187*/Y187*^ marrows was similar to that in mice transplanted with *Gmds^+/+^* cells, there was a significant reduction in the number of Gmds^Y187*/Y187*^-derived DP cells in the recipients (**Figure 4A**). There was a trend towards reduced numbers of CD4SP and CD8SP cells in the thymus of recipient mice, but the difference was not statistically significant (**Figure 4A**). Analysis of the spleen of recipient mice demonstrated that the absolute numbers of Gmds^Y187*/Y187*^-derived total T cells, CD4^+^ and CD8^+^ T cell subsets were comparable to *Gmds^+/+^*-derived cells (**Figure 4B**). The ratio of CD4 and CD8 T cells, and the CD44^lo^ naïve and CD44^hi^ memory T cell subsets within CD4^+^ and CD8^+^ cells in the spleen of *Rag1^-/-^* recipients engrafted with Gmds^Y187*/Y187*^ bone marrow cells were also comparable to those of recipients transplanted with *Gmds^+/+^* bone marrow cells (**Figure 4C**).

We also analyzed B cell development in the bone marrow and spleen of *Rag1^-/-^* recipient mice transplanted with Gmds^Y187*/Y187*^ bone marrow and found that B cell subsets were present in normal numbers compared to animals engrafted with *Gmds^+/+^* cells (**Figure 4D, E**). Interestingly, there was an increase in the percentage of SSC^hi^FSC^hi^ myeloid cells in the spleen of recipient mice engrafted with Gmds^Y187*/Y187*^ bone marrow cells (**Figure 4F**). Apart from the differences in immunological phenotypes, there was no apparent morbidity in *Rag1^-/-^* mice that were transplanted with Gmds^Y187*/Y187*^ bone marrow cells alone.

**Figure 5:**
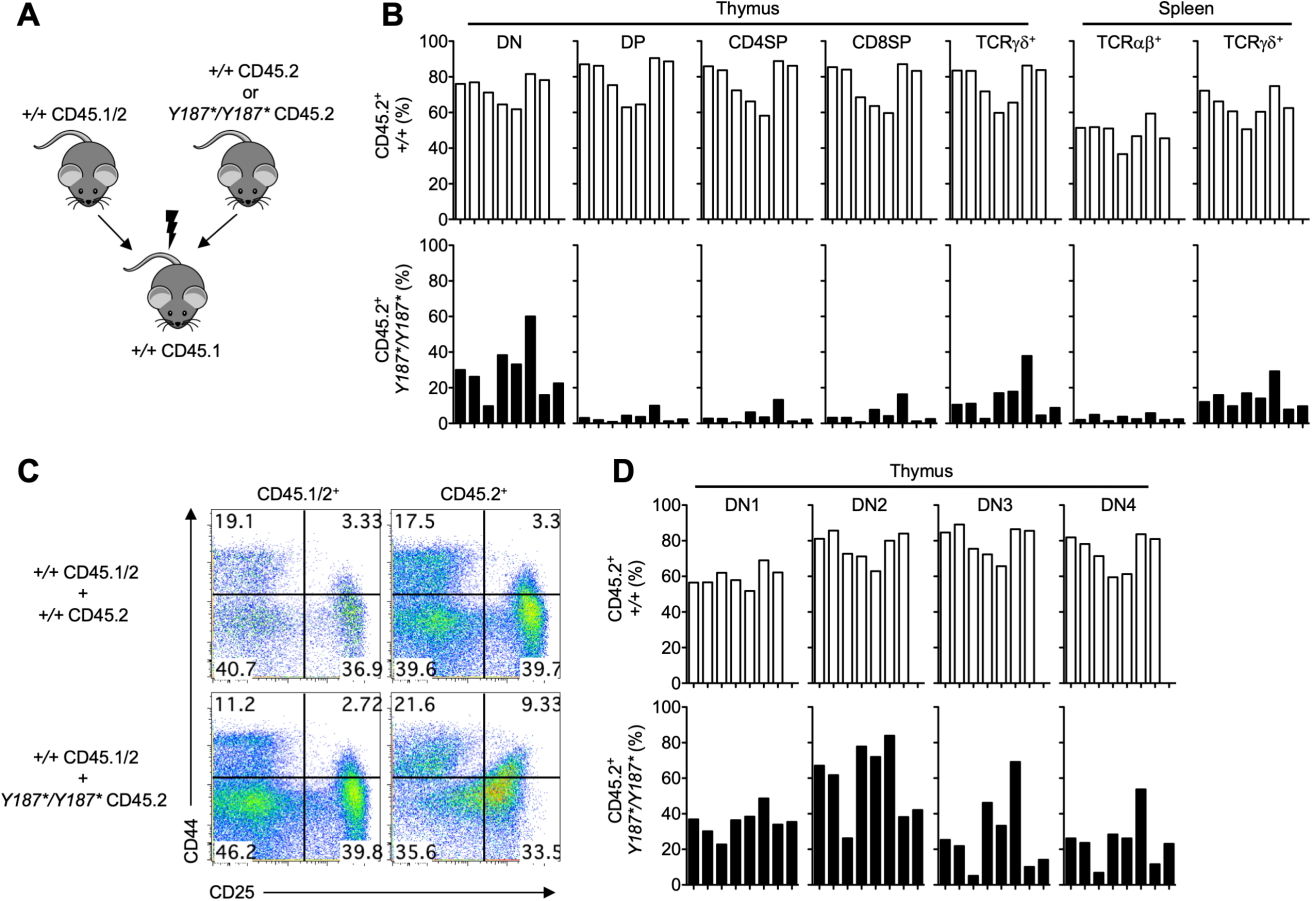
GMDS acts cell-intrinsically to regulate T cell development. **(A)** Bone marrow cells from either CD45.2 *Gmds^+/+^* or CD45.2 Gmds^Y187*/Y187*^ mice were mixed with CD45.1/2 *Gmds^+/+^* and transferred into irradiated CD45.1 *Gmds^+/+^* animals and analyzed by flow cytometry 10 weeks post-transplantation. **(B)** The graphs show the percentage of *Gmds^+/+^*- and Gmds^Y187*/Y187*^-derived CD45.2^+^ T cell subsets in the thymus and spleen of recipient mice. **(C)** Representative flow cytometric analysis of CD44 vs CD25 expression on CD45.1/2^+^ and CD45.2^+^ cells in the thymus of recipient mice. **(D)** The graphs show the percentage of *Gmds^+/+^*- and Gmds^Y187*/Y187*^-derived CD45.2^+^ DN cell subsets in the thymus of recipient mice. Each bar represents an individual recipient mouse.

In order to further assess whether GMDS controls T cell development in a cell-autonomous manner, we also analyzed the development and persistence of different immune cell types in a cohort of mixed bone marrow chimeras. The chimeras were established with a 1:1 ratio of bone marrow cells from *Gmds^+/+^* (CD45.2) or Gmds^Y187*/Y187*^ (CD45.2) and *Gmds^+/+^* (CD45.1/2), which were injected into sublethally irradiated *Gmds^+/+^* (CD45.1) recipients (**Figure 5A**). Consistent with our findings presented above, we found a profound developmental block at the DN stage of T cell development, as evidenced by a reduction in the proportion of Gmds^Y187*/Y187*^-derived DP, CD4SP and CD8SP T cells in the thymus of the chimeric recipients (**Figure 5B**). As a result of a defect in early development, Gmds^Y187*/Y187*^-derived T cells failed to accumulate in the spleen of irradiated recipients (**Figure 5B**). The percentage of Gmds^Y187*/Y187*^-derived TCRγδ cells was also reduced in the thymus and spleen of recipient mice, but the reduction was less compared to TCRαβ T cell subsets (**Figure 5B**). Analysis of different DN subsets revealed that there were relatively fewer Gmds^Y187*/Y187*^-derived DN3 and DN4 cells compared to DN1 and DN2 cells (**Figures 5C, D**). Interestingly, we again observed a reduced expression of CD25 on DN thymocytes derived from Gmds^Y187*/Y187*^ bone marrow but not on *Gmds^+/+^* T cells developing in the same animals, demonstrating that this is a cell-intrinsic phenotype and not secondary to any cell extrinsic alteration (**Figure 5C**).

We next tested the percentages of other immune cell types and found that B cells in the bone marrow and B cell subsets in the spleen of recipient mice transplanted with Gmds^Y187*/Y187*^ cells were broadly comparable to those transplanted with *Gmds^+/+^* bone marrow cells (**Supplementary Figure 4A**). Similarly, the percentage of Gmds^Y187*/Y187*^-derived CD11b^+^Gr1^-^, CD11b^+^Gr1^int^ and CD11b^+^Gr1^hi^ cells in the spleen of recipients was comparable to those transplanted with *Gmds^+/+^* cells (**Supplementary Figure 4B**). Interestingly, there was a decrease in the percentage of Gmds^Y187*/Y187*^-derived NK1.1^+^ Natural Killer (NK) cells compared to other cell types in the spleen of recipient mice (**Supplementary Figure 4B**).

These results were confirmed in a second cohort of mixed bone marrow chimeras but using irradiated immunocompromised (*Rag1^-/-^*) mice as recipients (**Supplementary Figures 5A–C**). Collectively, these findings demonstrate that the GMDS^Y187*^ mutation acts cell intrinsically to regulate T cell development *in vivo* and is dispensable for B cell development.

## 4 Discussion

The role of glycosylation in various biological processes has been extensively studied by various genetic manipulation experiments. In the present study, we examined a mouse strain with an *N*-ethyl-*N*-nitrosourea-induced point mutation in the gene encoding GMDS, which catalyzes the first step of GDP-fucose synthesis that is eventually used as a substrate for fucosylation. We report that the GMDS^Y187*^ mutation in mice resulted in growth retardation and a T cell defect due to a developmental block in T cell development in the thymus, which was more pronounced when Gmds^Y187*/Y187*^ cells had to compete with *Gmds^+/+^* cells in mixed chimeras, while other immune cell subsets remained largely unaffected. The GMDS^Y187*^ mutation introduces a premature STOP codon, resulting in a null allele and is likely to cause a loss of fucosylation activity. Thus, our results provide the first evidence of how a mutation in *Gmds* can affect T cell development in mice.

Fucosylation of glycans plays a critical role in the immune system (10, 34). Fucosylation on the Fc region of IgG1 antibodies has been of great interest as it causes a significant reduction in binding to its receptor, FcγRIIIa (CD16a), thus lack of fucosylation increases antibody-dependent cellular cytotoxicity (21, 22). This is particularly important for the application of cancer treatments, as non-fucosylated monoclonal antibodies can be used for therapeutic purposes, including cancer (35). Both FX- and GMDS-deficient Chinese hamster ovary host cells have been generated and proposed for use in the production of afucosylated therapeutic mAbs (36). In addition, a role for fucosylation in macrophage polarization and function has also been reported (37). Hematopoietic stem cells from fucosylation-deficient FX^-/-^ and Mx-Cre/Pofut1^F/F^ mice yielded increased numbers of CD11b^+^Gr1^+^ granulocytes in the periphery, suggesting a role for fucosylation in lymphoid and myeloid lineage commitment (13, 38). Core fucosylation has been shown to control T cell development via TCR signaling and T cell activation (24–26). It also attenuates PD-1 expression, thereby enhancing anti-cancer immune responses of T cells (27, 28). In addition to these studies, we demonstrated that GMDS with a previously unknown function in the immune system plays a critical role in T cell development, extending our understanding of the role of fucosylation in the immune system.

A key question arising from our results is how the mutation causes the T cell defect in Gmds^Y187*/Y187*^ mice. One possible explanation is a failure to produce the substrate (GDP-fucose) that is ultimately used to fucosylate targets in cellular signaling pathways that are important for T cell development. One example is the Notch signaling, which plays non-redundant roles in both invertebrates and vertebrates. Dysregulation of Notch signaling has been implicated in some developmental disorders and cancers. There are four Notch receptors (Notch 1-4) and five ligands (Jagged1 (Jag1) and Jag2 and delta-like 1 (Dll1), Dll3 and Dll4) in mammals (39). It has been well established that glycosylation of Notch receptors on their Epidermal Growth Factor (EGF)-like repeats in the extracellular domain regulates the ligand-receptor interactions by increasing or decreasing Notch activity (40). For example, Notch1 is *O*-fucosylated by POFUT in its serine or threonine residues within its EGF domains (41). Interestingly, defects in the genes encoding enzymes involved in GDP-fucose synthesis or transfer of GDP-fucose to the Golgi have been implicated in Notch-dependent lymphoid and myeloid cell development in the immune system (13, 34, 38). Therefore, it is plausible that the phenotype observed in Gmds^Y187*/Y187*^ mice deficient in *de novo* GDP-fucose synthesis could be due to dysregulation of the Notch signaling pathway, ultimately causing the T cell defect. However, as the focus of this study is to characterize the cellular process that is disrupted by the GMDS^Y187*^ mutation, further in-depth studies are needed to define the molecular consequences of the mutation.

Consistent with the above notion, there are remarkable similarities between Gmds^Y187*/Y187*^ mice and *Notch1* ^12f/12f^ animals, in which the *O*-fucose glycan cannot be attached to the EGF12 domain in Notch1 (42). First, our observation that the GMDS^Y187*^ mutation acts cell intrinsically to control T cell development in the thymus recapitulates the phenotype of *Notch1* ^12f/12f^ animals (42). In both cases, the number of thymocytes is reduced by almost 50% (42). Mice with a single copy of *Notch1* ^12f^ appeared normal as Gmds^Y187*/+^ animals, and the T cell deficiency in both deficient mice was due to a partial block at an early stage of T cell development in the thymus (42). This observation is also consistent with the finding that DP cells are more dependent on core fucosylation than DN cells (26). Second, the number of T cells in the spleen is unaffected in *Notch1* ^12f/12f^ and intact Gmds^Y187*/Y187*^ mice, suggesting that T cell emigration to the spleen is normal (42). This is also the case in mice with induced inactivation of *Notch1* (43). Third, similar to *Notch1* ^12f/12f^ animals (42), the T/B lineage decision appears to be unaffected by the GMDS^Y187*^ mutation, as evidenced by normal B cell development in the bone marrow and spleen in intact and chimeric recipient animals. However, the normal T/B lineage decision differs from the data obtained from Notch1-deficient mice, which show the existence of a B cell population in the thymus and a block at the DN1 to DN2 stage of T cell development in the thymus (43). Moreover, DN thymocytes from Gmds^Y187*/Y187*^ mice had significantly reduced surface expression of CD25, which is proposed to be positively regulated by Notch activation at the DN2 stage of T cell development (44–46). Taken together, these findings support the idea that the T cell phenotype observed in Gmds^Y187*/Y187*^ mice may be due to defective Notch receptor and ligand interactions or Notch signaling resulting from a lack of GDP-fucose and proper fucosylation. An observation that supports this notion is that Notch is not activated in FX^-/-^ mice, which are also deficient in the production of GDP-fucose (47, 48).

Despite these similarities, there are some inconsistent phenotypes in Gmds^Y187*/Y187*^ and Notch- and fucosylation-deficient animals. Notch2 deficiency in B cells resulted in impaired marginal zone (MZ) B cell formation in the spleen (49, 50) as seen in *Pofut1* ^-/-^ mice (38). However, Gmds^Y187*/Y187*^ bone marrow cells were able to give rise to B cell subsets, including MZ B cells, in the bone marrow and spleen of irradiated immunodeficient recipients, even in the presence of competition. Moreover, it has been reported that *Fut8* ^-/-^ mice have reduced numbers of pre-B cells due to a defect in the assembly and intracellular signaling of the pre-BCR resulting from defective core fucosylation of μ heavy chains (51). Nevertheless, our analysis revealed largely normal early B cell development in the bone marrow. Thus, the intact development of MZ B and pre-B cells in Gmds^Y187*/Y187*^ mice suggests that not all Notch activities are affected by the GMDS^Y187*^ mutation. Alternatively, there may be other pathways in addition to the Notch signaling pathway that can contribute to the T cell phenotype in Gmds^Y187*/Y187*^ mice. It should also be noted that the salvage pathway in Gmds^Y187*/Y187*^ mice can partially compensate for the loss of GDP-fucose synthesis, which may be sufficient for the development of some cell types that are less dependent on fucosylation. This may also explain the milder phenotype observed in bone marrow chimeras where *Gmds^+/+^* recipient mice were transplanted with Gmds^Y187*/Y187*^ bone marrow cells (**Figure 4**), compared to intact Gmds^Y187*/Y187*^ mice, which lack GMDS activity in all cells.

Aberrant fucosylation has been observed in many diseases, and dysregulation of the fucosylation process has also been reported to be associated with developmental abnormalities and several types of cancer. For example, a mutation in *Gmds* of the colon cancer cell line HCT116 resulted in loss of fucosylation and escape of cancer cells from NK cell-mediated immune surveillance (52). The loss of fucosylation observed in the cell line strongly suggests that the premature STOP codon in our mice will also abolish cellular fucosylation, however confirmation of this requires further experiments. Several mutations in *GMDS* have been identified in tissue samples from patients with colorectal cancer (53). Similarly, FUT4 has been proposed as a marker for breast cancer (54), and expression of FUT8 is positively correlated with migration and invasiveness of breast cancer cells (55, 56) and lung cancer (57). Thus, although we have focused our analysis here on the immunological phenotypes, the growth retardation and postnatal mortality observed in intact Gmds^Y187*/Y187*^ mice could be attributed to the other developmental mechanisms regulated by fucosylation, since loss of T cells does not affect postnatal development and growth (58). Consistent with this notion, loss of fucosylation in FX^-/-^ and *Notch1* ^12f/12f^ resulted in embryonic lethality, reduced weight gain and postnatal survival as observed in Gmds^Y187*/Y187*^ mice (12, 42). These findings suggest a broader effect of cellular fucosylation on various biological processes, but these are beyond the scope of this study and require further analysis.

In conclusion, our results provide an insight into the role of GMDS in the development of murine T cells. The T cell deficiency caused by the GMDS^Y187*^ mutation was more pronounced in irradiated recipients of mixed chimeras when there was competition, suggesting a critical involvement of fucosylation in T cell biology.

## Data Availability

The raw data supporting the conclusions of this article will be made available by the authors, without undue reservation.
