# Supplementary material for: Essential cell-intrinsic requirement for GMDS in T cell development
Source: Front Immunol. 2025 Jun 26;16:1598923. doi: 10.3389/fimmu.2025.1598923 (PMC12241148; doi:10.3389/fimmu.2025.1598923)
Supplement: Supplementary file 1 [file DataSheet1.pdf]

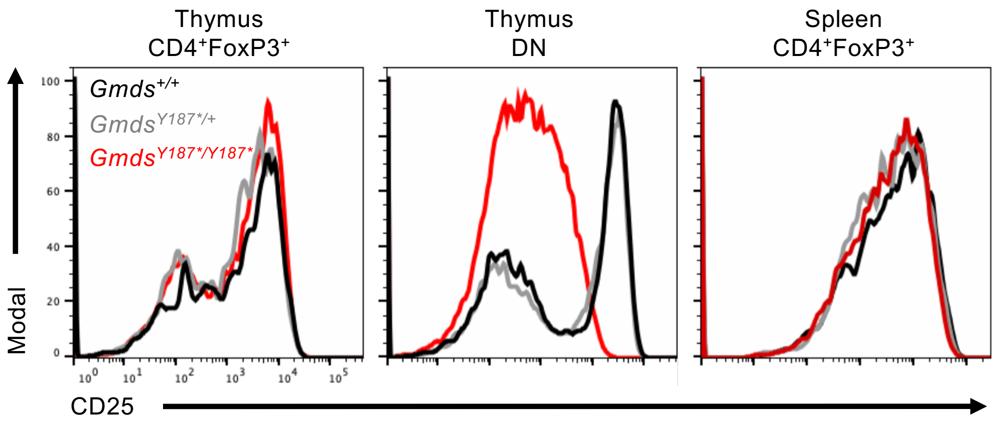

SUPPLEMENTARY FIGURE 1 Normal CD25 expression on CD4$^+$FoxP3$^+$ T cells in the thymus and spleen of *Gmds*$^{Y187*/Y187*}$ mice. The overlay histograms show CD25 expression on thymic CD4$^+$FoxP3$^+$ and DN T cells, and splenic CD4$^+$FoxP3$^+$ T cells from *Gmds*$^{+/+}$ (black line), *Gmds*$^{Y187*/+}$ (grey line) and *Gmds*$^{Y187*/Y187*}$ (red line).

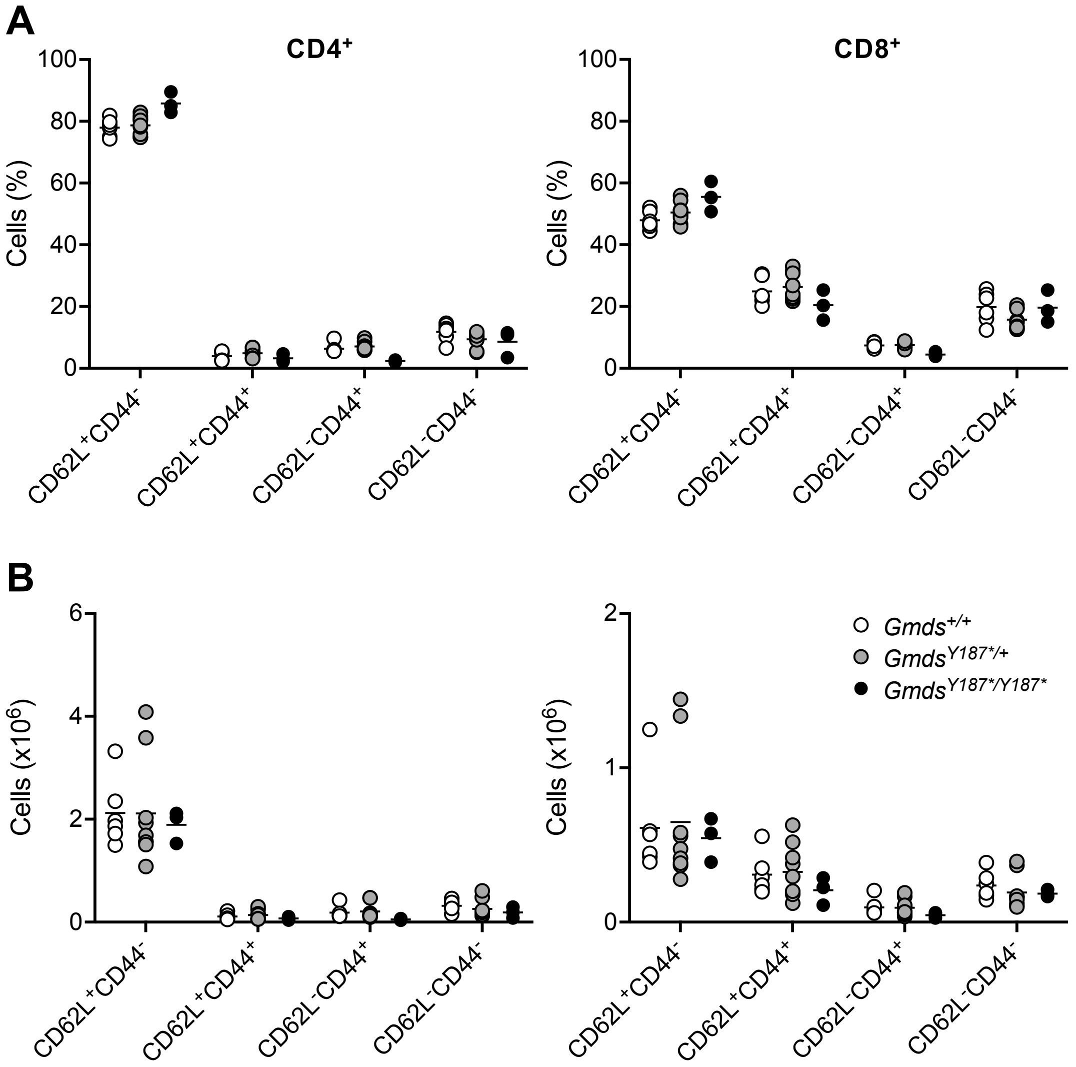

SUPPLEMENTARY FIGURE 2 Presence of T cell subsets in the spleen of *Gmds*$^{Y187*/Y187*}$ mice at 3 weeks of age. The graphs show **(A)** the percentage and **(B)** the absolute numbers of T cell subsets in *Gmds*$^{+/+}$, *Gmds*$^{Y187*/+}$ and *Gmds*$^{Y187*/Y187*}$ mice at 3 weeks of age. Each symbol represents an individual mouse, and n=6 for *Gmds*$^{+/+}$, n=9 for *Gmds*$^{Y187*/+}$ and n=3 for *Gmds*$^{Y187*/Y187*}$ pooled from two independent experiments.

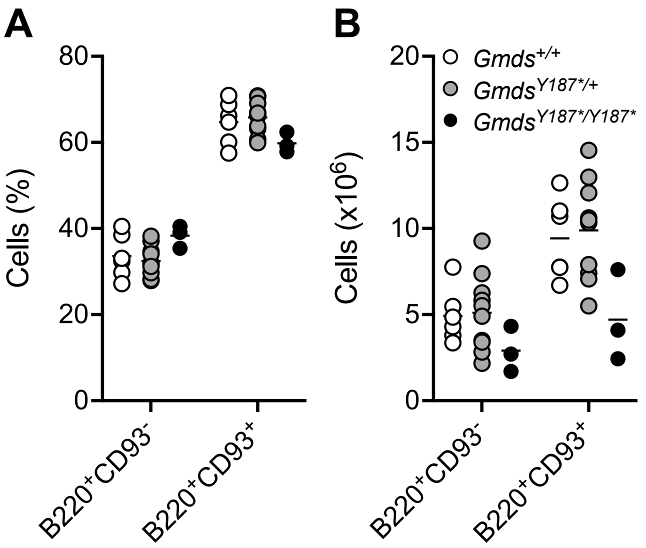

SUPPLEMENTARY FIGURE 3 Presence of mature and immature B cells in the spleen of *Gmds*$^{Y187*/Y187*}$ mice at 3 weeks of age. The graphs show **(A)** the percentage and **(B)** the absolute numbers of mature (B220^{+}CD93^{-}) and immature (B220^{+}CD93^{+}) B cells in *Gmds*$^{+/+}$, *Gmds*$^{Y187*/+}$ and *Gmds*$^{Y187*/Y187*}$ mice at 3 weeks of age. Each symbol represents an individual mouse, and n=6 for *Gmds*$^{+/+}$, n=10 for *Gmds*$^{Y187*/+}$ and n=3 for *Gmds*$^{Y187*/Y187*}$ pooled from two independent experiments.

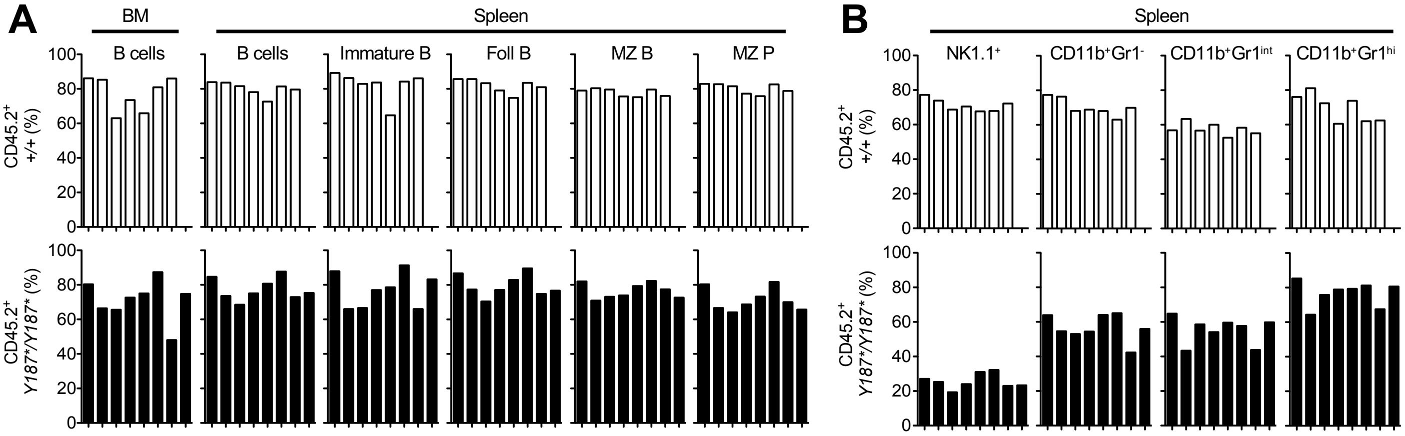

SUPPLEMENTARY FIGURE 4 Normal *Gmds*$^{Y187*/Y187*}$-derived B and myeloid cells in mixed chimeric recipients. Bone marrow cells from either CD45.2 *Gmds*$^{+/+}$ or CD45.2 *Gmds*$^{Y187*/Y187*}$ mice were mixed with CD45.1/2 *Gmds*$^{+/+}$ and transferred into irradiated CD45.1 *Gmds*$^{+/+}$ animals and analyzed by flow cytometry 10 weeks post-transplantation as in Figure 5A. **(A)** The graphs show the percentage of *Gmds*$^{+/+}$- and *Gmds*$^{Y187*/Y187*}$-derived CD45.2^{+} total B cells in the bone marrow, and total B and different B cell subsets in the spleen of recipient mice. **(B)** The graphs show the percentage of *Gmds*$^{+/+}$- and *Gmds*$^{Y187*/Y187*}$-derived CD45.2^{+} NK1.1^{+}, CD11b^{+}Gr1^{-}, CD11b^{+}Gr1^{int} and CD11b^{+}Gr1^{hi} cells in the spleen of recipient mice. Each bar represents an individual recipient mouse.

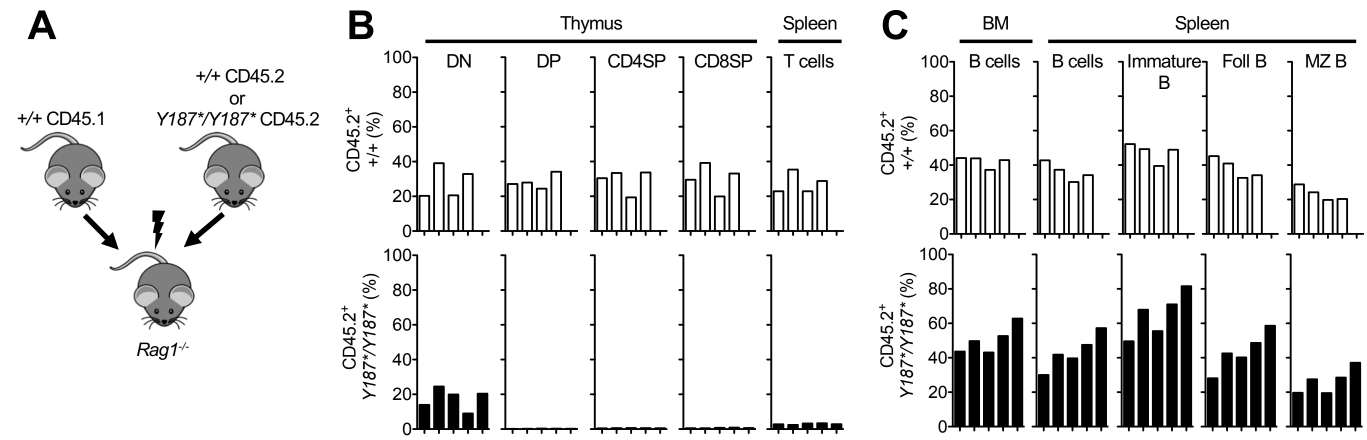

SUPPLEMENTARY FIGURE 5 GMDS acts cell-intrinsically to regulate T cell development. **(A)** Another set of mixed chimeras was generated by mixing bone marrow cells from either CD45.2 *Gmds*$^{+/+}$ or CD45.2 *Gmds*$^{Y187*/Y187*}$ mice with CD45.1 *Gmds*$^{+/+}$ and transferring into irradiated *Rag1*$^{-/-}$ animals and analyzed by flow cytometry 158 days post-transplantation. **(B)** The graphs show the percentage of *Gmds*$^{+/+}$- and *Gmds*$^{Y187*/Y187*}$-derived CD45.2^{+} T cell subsets in the thymus and spleen of recipient mice. **(C)** The graphs show the percentage of *Gmds*$^{+/+}$- and *Gmds*$^{Y187*/Y187*}$-derived CD45.2^{+} total B cells in the bone marrow, and total B and different B cell subsets in the spleen of recipient mice. Each bar represents an individual recipient mouse.
